# Traditional Chinese Medicine Use in the Treatment of Acute Heart Failure in Western Medicine Hospitals in China: Analysis From the China PEACE Retrospective Heart Failure Study

**DOI:** 10.1161/JAHA.119.012776

**Published:** 2019-07-31

**Authors:** Yuan Yu, Erica S. Spatz, Qi Tan, Shuling Liu, Yuan Lu, Frederick A. Masoudi, Wade L. Schulz, Harlan M. Krumholz, Jing Li, the China PEACE Collaborative Group

**Affiliations:** ^1^ The China PEACE Collaborative Group: NHC Key Laboratory of Clinical Research for Cardiovascular Medications National Clinical Research Center of Cardiovascular Diseases, Fuwai Hospital National Center for Cardiovascular Diseases Chinese Academy of Medical Sciences and Peking Union Medical College Beijing China; ^2^ Central China Subcenter of the National Center for Cardiovascular Diseases Henan People's Republic of China; ^3^ Center for Outcomes Research and Evaluation Yale–New Haven Hospital New Haven CT; ^4^ Section of Cardiovascular Medicine Yale School of Medicine New Haven CT; ^5^ Department of Laboratory Medicine Yale School of Medicine New Haven CT; ^6^ Department of Internal Medicine Yale School of Medicine New Haven CT; ^7^ Department of Biostatistics Yale School of Public Health Yale University New Haven CT; ^8^ Department of Health Policy and Management Yale School of Public Health Yale University New Haven CT; ^9^ Division of Cardiology University of Colorado Anschutz Medical Campus Aurora CO

**Keywords:** China, heart failure, hospital variation, traditional Chinese medicine, Heart Failure, Treatment, Mortality/Survival, Complications

## Abstract

**Background:**

Traditional Chinese medicine (TCM) is used in the treatment of many conditions, including heart failure (HF), although it is not well characterized.

**Methods and Results:**

We conducted a retrospective analysis of TCM use in a random sample of hospitalizations for HF within a random sample of Western medicine hospitals in China in 2015 using data from the China PEACE 5r‐HF (China Patient‐Centered Evaluative Assessment of Cardiac Events 5 Retrospective Heart Failure Study). We describe the frequency of TCM use and its association with patient characteristics, in‐hospital use of evidence‐based therapies, and hospital characteristics using hierarchical logistic regression models. Finally, we assessed risk‐adjusted in‐hospital bleeding and mortality. Among 10 004 patients hospitalized with HF (median age, 73 years; 48.9% women) from 189 hospitals, 74.7% received TCM (83.3% administered intravenously). The most commonly used agent was Salvia miltiorrhiza (51.2%). Patients with coronary artery disease (odds ratio [OR], 1.73; 95% CI, 1.53–1.95) or stroke (OR, 1.32; 95% CI, 1.15–1.51) were more likely to receive TCM; there was no correlation with evidence‐based therapy use. Nearly all hospitals (99.4%) used TCM, with substantial variation across hospitals (median OR, 3.29; 95% CI, 2.82–3.76). In‐patient bleeding (OR, 1.39; 95% CI, 1.03–1.88) and mortality (OR, 1.36; 95% CI, 1.04–1.79) were higher with Salvia miltiorrhiza, although not with other TCMs.

**Conclusions:**

In a nationally representative sample of patients hospitalized with acute HF in China, three fourths received TCM. Nearly all hospitals used TCM, although use varied substantially by hospital. Although TCM was not used in lieu of evidence‐based therapies for HF, we found a signal for harm with the most commonly used TCM.

**Clinical Trial Registration:**

URL: https://www.clinicaltrials.gov. Unique identifier: NCT02877914.

**Clinical Perspective**

**What Is New?**

- Traditional Chinese medicine (TCM) was used in nearly every Western hospital in China for the treatment of acute decompensated heart failure, although the extent of use varied substantially by hospital.
- Although TCM was not used in lieu of evidence‐based therapies for heart failure, we found a signal for harm with the most widely used TCM.

**What Are the Clinical Implications?**

- Given the frequency of hospitalizations for heart failure in China and the magnitude of use of TCM for this condition, there is an urgent need for further research into the safety and efficacy of TCM, along with standardized approaches for its use in clinical practice.

## Introduction

Traditional Chinese medicine (TCM) is commonly used as a complement to evidence‐based therapies for acute illnesses in China.^1^ A recent study of acute myocardial infarction found that more than half of patients in Chinese Western medicine hospitals received intravenous TCM as short‐term therapy in 2011.^2^ Prior studies suggest that TCM is also frequently used for the management of heart failure (HF), although these studies have been limited to specific provinces or cities in China^3^, ^4^, ^5^ and questions remain about the pervasiveness of TCM in the management of patients hospitalized with HF in China, with implications for clinical practice, research, and policy.

Despite systematic reviews^6^, ^7^, ^8^, ^9^ showing inconclusive benefits of TCM in the treatment of HF and a lack of support for TCM in Chinese guidelines,^10^, ^11^ the limited existing data suggest that TCM is increasingly being used in the management of HF.^12^ TCM use may be driven by wide‐scale availability, even in Western medicine hospitals; small studies suggesting benefits in symptoms, hemodynamics, and outcomes^13^, ^14^, ^15^, ^16^, ^17^, ^18^; and a cultural desire to preserve an ancient medical tradition. Yet, as the Chinese health system strives to become a high‐performing, evidence‐based system, an understanding of the extent of TCM use, the patient factors associated with its use, the extent of hospital‐level variation in use, and whether TCM is used as a substitute for proven therapies for HF would provide the basis for understanding how the quality of HF care might be improved.

Accordingly, we used data from a nationally representative study of patients with acute decompensated HF admitted to Western medicine hospitals throughout China in 2015 to investigate current use patterns of TCM. Collection of TCM data was part of the China PEACE 5r‐HF (China Patient‐Centered Evaluative Assessment of Cardiac Events 5 Retrospective Heart Failure Study).^19^ Our aims herein were as follows: (1) to describe practice patterns in the use of TCM during HF hospitalization, including type, combination, and duration; (2) to assess patient characteristics associated with TCM use; (3) to describe hospital variation in use of TCM; and (4) to examine the association of TCM with inpatient bleeding and mortality in an exploratory analysis. Given the frequency of hospitalizations for HF in China, this study can provide important data to guide next steps for determining the efficacy and safety of TCM for acute decompensated HF.

## Methods

The study materials have been made available to other researchers for purposes of replicating the procedure.^19^ It is our goal to share the China PEACE 5r‐HF prospective study data; however, at this time, we are unable to do so.

### Study Design

The China PEACE 5r‐HF used a 2‐stage random sampling design to create a nationally representative sample of hospital admissions for HF, with the goal of assessing quality and outcomes of patients hospitalized with HF in 2015. The study design has been described previously.^19^ Briefly, we first identified a nationally representative cohort of 189 Western medicine hospitals (a phrase used in China to designate hospitals that are based in Western or conventional therapies, distinguishing them from TCM hospitals). Second, we used systematic random sampling procedures to select 10 004 patients hospitalized for HF in 2015 from the local hospital database of each sampled hospital. As previously described, centralized data abstraction of charts using standardized definitions was assessed for accuracy and quality.^19^

Informed consent was waived for patients not involved in the recruitment or conduct of the study. The Central Ethics Committee at the Chinese National Center for Cardiovascular Diseases and Yale University approved the study. All collaborating hospitals accepted central ethics approval, except for 15 hospitals, which obtained local approval by their internal ethics committees.

### Classification of TCM

Herbal medicines, including the name, route of administration, and timing of use in the course of hospitalization, were abstracted from the medical record. Use of nonpharmacological TCM (eg, qigong and acupuncture) is limited in Chinese Western medicine hospitals, if at all used, and therefore was not abstracted. Herbal medicines were classified into groups on the basis of the primary active ingredient. If an herbal medicine included >1 active ingredient, we classified it according to the primary active ingredient, as previously reported.^2^, ^3^ We focused on the most commonly used TCMs among patients with HF (defined as being used in >5% of patients in the sample): Salvia miltiorrhiza, Panax notoginseng, Gingko, Radiax astragali, and Safflower (Table S1). Less frequently used agents were combined into a category of other TCM. The duration of TCM use during hospitalization was based on physicians’ orders, counted in days, based on whether any TCM was prescribed on each day of hospitalization.

### Statistical Analysis

We describe the frequency of oral and intravenous TCM use among patients admitted for HF, including TCM type, timing of administration (during hospitalization or at discharge), and duration of each TCM use during hospitalization. For the evaluation of medications at discharge, we excluded patients who died or withdrew care during hospitalization. We examined pairwise correlations to assess commonly used combinations of the TCMs during hospitalization; specifically, we calculated the frequency of use of each pair of the 5 TCMs and calculated the Pearson correlation coefficient to examine the association between each TCM pair. In addition, we compared patient‐ and hospital‐level characteristics by the use of any TCM during hospitalization.

To identify factors associated with TCM, we used hierarchical logistic regression models with backward stepwise selection of covariates that were significant at the 0.05 level, using a random effect at the hospital level to account for the patients’ clustering within hospitals. We selected explanatory variables, on the basis of clinical judgment and review of the literature, hypothesized to be associated with TCM use, including demographics, clinical characteristics, clinical care management, and hospital‐level characteristics. To assess whether TCM was used in lieu of guideline‐based therapies or recommended diagnostic tests, we tested the association of TCM use with guideline‐based therapies in unadjusted and multivariable models. We transformed continuous variables into categorical variables, according to clinically meaningful cutoff values, and then created dummy variables. For variables with >1% of data missing, we created a dummy variable indicating missing and included it with the original variable in the adjusted models. Patients with a length of stay of ≤24 hours were excluded. We report odds ratios (ORs) with 95% CIs.

At the hospital level, use of TCM was calculated for each hospital by determining the proportion of patients who received TCM during hospitalization. Hospitals with <25 total admissions were excluded from this analysis. To estimate the variability in hospital‐level TCM use, we calculated the median OR from the fully adjusted hierarchical model, which represents the average likelihood of a statistically identical patient receiving TCM at one random hospital versus another.^20^

In exploratory analyses, we compared outcomes of in‐hospital bleeding and in‐hospital mortality/withdrawal of care among patients receiving any TCM. Withdrawal from treatment is a common disposition among patients deemed to be actively dying, but who do not want to die in the hospital; this disposition status was adjudicated by clinicians in the coordinating study center. The outcome of in‐hospital mortality or treatment withdrawal is used as a quality measure for hospitals by the Chinese government. We also assessed in‐hospital bleeding given the antiplatelet effects of many of the TCMs and the potential for bleeding, especially if combined with other antiplatelet or anticoagulant use.^21^ Bleeding was defined as a documented bleeding event in medical records or a decrease in hemoglobin of ≥3 g/dL. We adjusted variables hypothesized to be associated with in‐hospital bleeding or mortality, including demographics, clinical characteristics, clinical care management (including antiplatelet and anticoagulant agents), and hospital‐level characteristics. In addition, we evaluated in‐hospital outcomes stratified by the duration of Salvia miltiorrhiza therapy on the basis of the distribution of days used into the following: 1, 2 to 9, and >9 days. We also evaluated in‐hospital outcomes stratified by in‐hospital use of antiplatelet or anticoagulant agents and by Salvia miltiorrhiza.

Statistical analysis was performed with SAS software, version 9.3.

## Results

### Use Patterns of TCM

**Figure 1:**
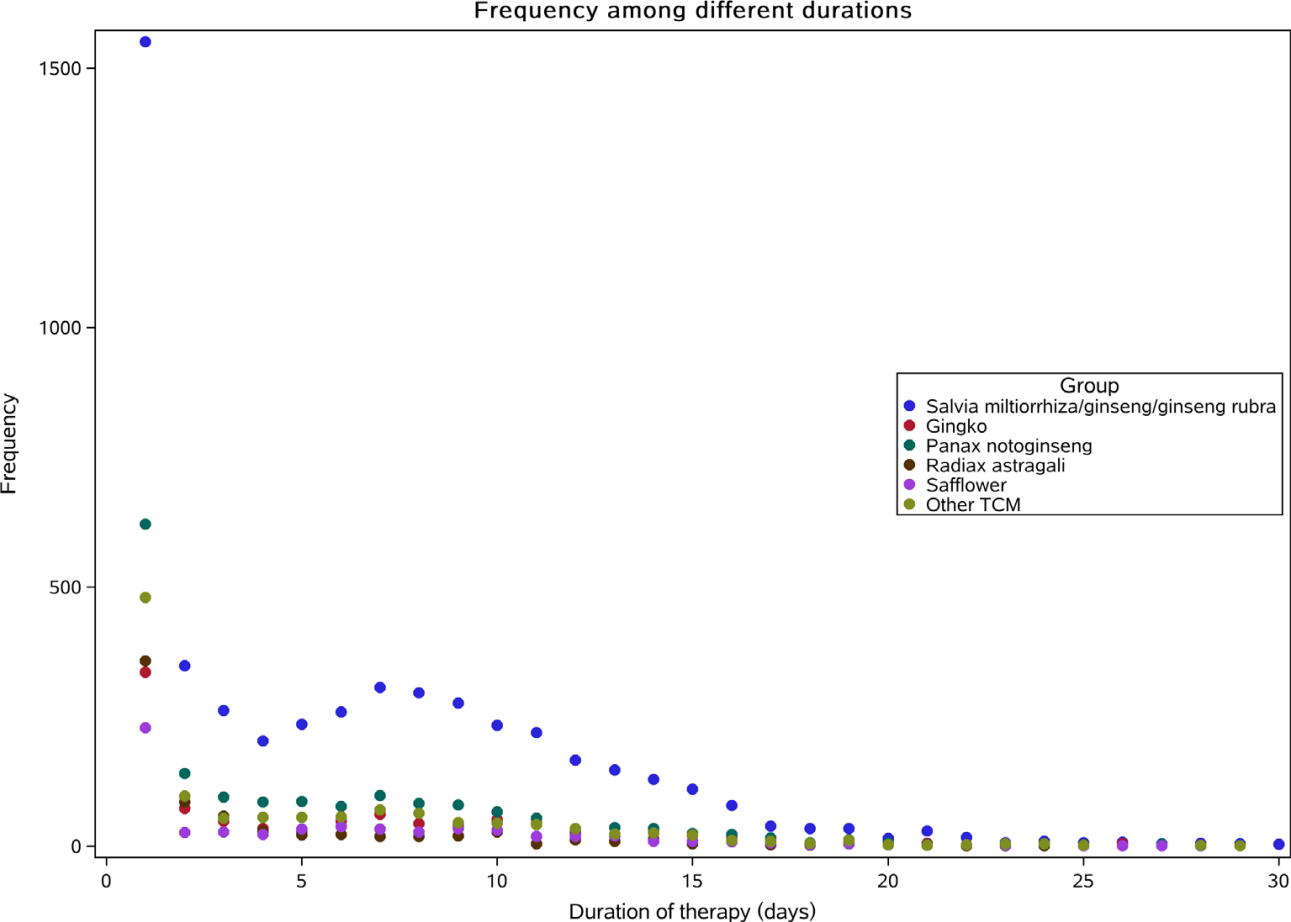
Duration of traditional Chinese medicine (TCM) use during heart failure hospitalization.

Among 10 004 patients admitted for HF, 74.7% received TCM during hospitalization, which was most often (83.3%) administered intravenously. Only 12.3% were prescribed TCM at discharge. During hospitalization, the most commonly used TCMs were Salvia miltiorrhiza (51.2%), followed by Panax notoginseng (17.1%), Gingko (9.7%), Radiax astragali (7.4%), and Safflower (6.3%). Nearly a quarter (24.2%) of patients received ≥2 types of TCM, with the most common combination being Salvia miltiorrhiza with either Panax notoginseng (8.2%) or Radiax astragali (4.4%) (Table 1). For patients who received TCM during hospitalization, the median duration for TCM use was 6 days (interquartile range [IQR], 1–11 days) and the median length of stay was 9 days (IQR, 6–13 days); 27.1% of patients received only 1‐day therapy of any TCM (Figure 1). Patients who received 1‐day therapy of Salvia miltiorrhiza accounted for 30.6% of patients who received Salvia miltiorrhiza; similar proportions of patients received 1‐day therapy for Panax notoginseng, Gingko, Radiax astragali, and Safflower (34.8%, 36.5%, 48.3%, and 36.8%, respectively). Most patients received TCM on the first day of admission, and the percentage of TCM use decreased markedly thereafter (Figure S1).

**Table 1:** TCM Use During Hospitalization for HF in Western Medicine Hospitals in China

Type of TCM	Salvia Miltiorrhiza, Ginseng, Ginseng Rubra	Panax Notoginseng	Gingko	Radiax Astragali	Safflower
Salvia miltiorrhiza, ginseng, ginseng rubra	5072 (51.2)	809 (8.2)	371 (3.7)	433 (4.4)	226 (2.3)
Panax notoginseng	…	1696 (17.1)	135 (1.4)	130 (1.3)	56 (0.6)
Gingko	…	…	962 (9.7)	81 (0.8)	53 (0.5)
Radiax astragali	…	…	…	730 (7.4)	65 (0.7)
Safflower	…	…	…	…	622 (6.3)

Data are given as number (percentage). HF indicates heart failure; TCM, traditional Chinese medicine.

### Characteristics of Patients and Hospitals Using TCM

Patient characteristics were compared between patients who received any TCM and patients who did not receive TCM (Table 2). The median age of patients was similar (73 [IQR, 65‐80] versus 73 [IQR, 63‐80] years, respectively). Patients receiving TCM were more likely to be women than those not receiving TCM (49.8% versus 46.3%; *P*=0.003). In addition, bivariate analyses revealed that in‐hospital tests differed between patients receiving TCM and patients who did not (Table 3). Patients receiving TCM were less likely to have brain natriuretic peptide or NT‐proBNP (N‐terminal pro‐B‐type natriuretic peptide) testing (53.7% versus 63.9%; *P*<0.001) or chest x‐ray or computed tomographic scan (74.1% versus 77.0%; *P*=0.004) compared with patients who did not receive TCM.

**Table 2:** Demographic and Clinical Characteristics Stratified by Use of TCM During Hospitalization

Characteristics	Overall		TCM		No TCM		*P* Value
	n	%	n	%	n	%	
All	9909	…	7400	74.7	2509	25.3	…
Demographics							
Age, median (IQR), y	73 (65–80)		73 (65–80)		73 (63–80)		0.0351
Age categories, y							
<55	890	9.0	625	8.4	265	10.6	0.0004
55–64	1536	15.5	1120	15.2	416	16.6	
65–74	2948	29.8	2240	30.3	708	28.2	
75–84	3492	35.2	2658	35.9	834	33.2	
≥85	1043	10.5	757	10.2	286	11.4	
Women	4845	48.9	3683	49.8	1162	46.3	0.0028
Medical insurance	9052	91.4	6773	91.5	2279	90.8	0.2852
Comorbidities							
Coronary artery disease	6013	60.7	4718	63.8	1295	51.6	<0.0001
Hypertension	5327	53.8	4012	54.2	1315	52.4	0.1171
Atrial fibrillation	3565	36.0	2704	36.5	861	34.3	0.0449
Cardiac valvular disease	3334	33.6	2416	32.6	918	36.6	0.0003
COPD or asthma	3023	30.5	2158	29.2	865	34.5	<0.0001
Dyslipidemia	5022	50.7	3686	49.8	1336	53.2	0.0029
Stroke/transient ischemic attack	1992	20.1	1545	20.9	447	17.8	0.0009
Diabetes mellitus	1977	20.0	1424	19.2	553	22.0	0.0024
Chronic renal insufficiency	1621	16.4	1100	14.9	521	20.8	<0.0001
Peripheral vascular disease	880	8.9	593	8.0	287	11.4	<0.0001
Cancer	354	3.6	235	3.2	119	4.7	0.0003
Anemia	2490	25.1	1804	24.4	686	27.3	0.0031
Clinical presentation							
Admission to ICU/CCU	549	5.5	352	4.8	197	7.8	<0.0001
Cardiogenic shock	21	0.2	16	0.2	5	0.2	0.8734
Chest pain	6409	64.7	4938	66.7	1471	58.6	<0.0001
Dyspnea at rest	5081	51.3	3699	50.0	1382	55.1	<0.0001
Orthopnea	2696	27.2	1956	26.4	740	29.5	0.0029
Dyspnea on exertion	4962	50.1	3765	50.9	1197	47.7	0.0061
Paroxysmal nocturnal dyspnea	1526	15.4	1131	15.3	395	15.7	0.5816
Jugular vein distension	2591	26.2	1911	25.8	680	27.1	0.3754
S3 present	16	0.2	9	0.1	7	0.3	<0.0001
Pulmonary rales present	5499	55.5	3966	53.6	1533	61.1	<0.0001
Hepatojugular reflux positive	536	5.4	420	5.7	116	4.6	<0.0001
Lower‐extremity edema	4963	50.1	3728	50.4	1235	49.2	0.6006
Heart rate, bpm							
<60	423	4.3	320	4.3	103	4.1	0.8432
60–99	6223	62.8	4652	62.9	1571	62.6	
≥100	3261	32.9	2427	32.8	834	33.2	
Systolic blood pressure, mm Hg							
<90	112	1.1	84	1.1	28	1.1	0.9970
90–139	5565	56.2	4156	56.2	1409	56.2	
≥140	4221	42.6	3153	42.6	1068	42.6	
Diastolic blood pressure, mm Hg							
<60	407	4.1	289	3.9	118	4.7	0.2135
60–89	6532	65.9	4893	66.1	1639	65.3	
≥90	2957	29.8	2210	29.9	747	29.8	
NYHA functional class							
II	1087	10.9	797	1.8	290	11.6	<0.0001
III	3980	40.2	3081	41.6	899	35.8	
IV	3060	30.9	2234	30.2	826	32.9	
Unrecorded	1782	18.0	1288	17.4	494	19.7	
GWTG‐HF risk score, median (IQR)	36 (32–41)		36 (32–41)		37 (32–41)		0.0177

Bpm indicates beats per minute; CCU, cardiovascular care unit; COPD, chronic obstructive pulmonary disease; GWTG‐HF, Get With The Guidelines–Heart Failure; ICU, intensive care unit; IQR, interquartile range; NYHA, New York Heart Association; TCM, traditional Chinese medicine.

**Table 3:** Clinical Management Stratified by Use of TCM During Hospitalization

Variable	Overall		TCM		No TCM		*P* Value
	n	%	n	%	n	%	
All	9909	…	7400	74.7	2509	25.3	…
Medication during hospitalization*							
ACEI (eligible: 1278)*	627	49.1	474	50.9	153	44.1	0.0301
ARB (eligible: 1278)*	276	21.6	175	18.8	101	29.1	<0.0001
ACEI or ARB (eligible: 1278)*	863	67.5	621	66.7	242	69.7	0.3023
β‐Blocker (eligible: 1345)*	790	58.7	574	58.8	216	58.5	0.9272
Aldosterone receptor antagonist (eligible: 1312)*	1146	87.6	836	87.6	310	86.6	0.6141
Anticoagulant (eligible: 1240)*	458	36.9	319	35.6	139	40.5	0.1054
Tests							
BNP/NT‐proBNP	5578	56.3	3974	53.7	1604	63.9	<0.0001
Chest x‐ray or CT scan	7415	74.8	5484	74.1	1931	77.0	0.0044
Echocardiogram	6436	65.0	4765	64.4	1671	66.6	0.0248
Ejection fraction, %							
Unknown	3634	36.7	2761	37.3	873	34.8	0.0238
<40	1379	13.9	1000	13.5	379	15.1	0.0465
≥40 and <50	1115	11.2	821	11.1	294	11.7	0.3933
≥50	3781	38.2	2818	38.1	963	38.4	0.7887
Laboratory data median (IQR)							
Blood urea nitrogen, mmol/L	7 (5–10)		7 (5–9)		7 (5–10)		<0.0001
Serum creatinine, μmol/L	0.95 (0.76–1.25)		0.95 (0.76–1.23)		0.98 (0.77–1.34)		<0.0001
Serum sodium, mEq/L	139.6 (136.5–142)		139.7 (136.7–142)		139.2 (136–142)		0.0507
Total cholesterol, mmol/L	4.0 (3.3–4.8)		4.0 (3.3–4.8)		4.0 (3.3–4.8)		0.0514
Glucose, mmol/L	6 (5.0–7.6)		5.9 (5–7.5)		6.1 (5.1–7.8)		0.0006
Hemoglobin, g/L	129 (113–143)		129 (114–143)		128 (112–144)		0.2436
Serum potassium, mmol/L	4 (3.6–4.4)		4 (3.6–4.4)		4 (3.6–4.4)		0.6995
Medication at discharge*							
ACEI* (eligible: 1261)	442	35.0	326	35.4	116	34.1	0.6728
ARB* (eligible: 1261)	209	16.6	129	14.0	80	23.5	<0.0001
ACEI or ARB* (eligible: 1261)	648	51.4	454	49.3	194	57.1	0.0144
β‐Blocker* (eligible: 1321)	609	46.1	438	45.6	171	47.5	0.5325
Aldosterone receptor antagonist* (eligible: 1289)	836	64.9	598	63.7	238	68.0	0.1489
Anticoagulant* (eligible: 1218)	54	4.4	25	2.8	29	8.6	<0.0001

ACEI indicates angiotensin‐converting enzyme inhibitor; ARB, angiotensin receptor blocker; BNP, brain natriuretic peptide; CT, computed tomographic; NT‐proBNP, N‐terminal pro‐B‐type natriuretic peptide; TCM, traditional Chinese medicine.

* Analysis among eligible patients with indications and without contraindications.

**Figure 2:**
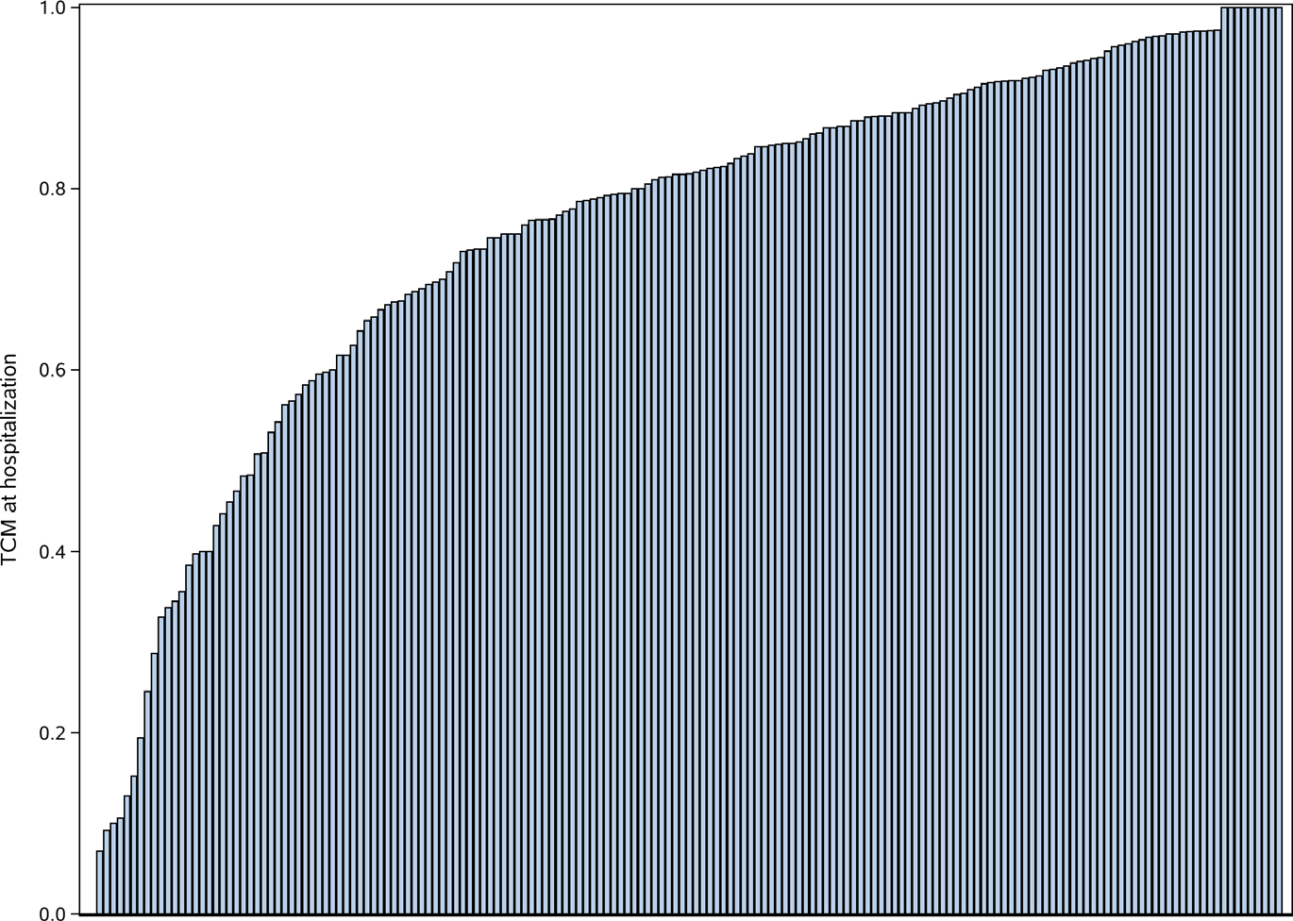
Variation of inpatient traditional Chinese medicine (TCM) use among participating hospitals.

**Figure 3:**
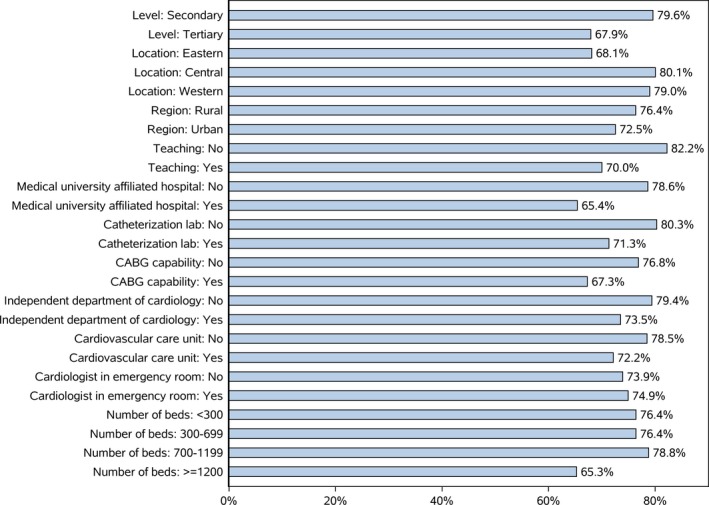
Traditional Chinese medicine use by hospital subtype. CABG indicates coronary artery bypass grafting.

Although nearly all the sampled hospitals (99.4%) used TCM in at least some patients with HF, there was significant variation in frequency of use across hospitals (Figure 2). The median frequency of hospital TCM use was 81.6% (range, 0%–100%; IQR, 67.2%–91.6%). We also observed variation in TCM use by hospital subtype (Figure 3). The median rate of TCM use was 86.7% (IQR, 76.6%–93.3%) in nonteaching hospitals compared with 78.6% (IQR, 59.3%–88.2%) in teaching hospitals; and it 84.2% (IQR, 69.8%–92.2%) in non–coronary artery bypass grafting hospitals compared with 74.8% (IQR, 46.9%–82.1%) in coronary artery bypass grafting hospitals.

### Patient and Hospital Characteristics Associated With TCM Administration

**Figure 4:**
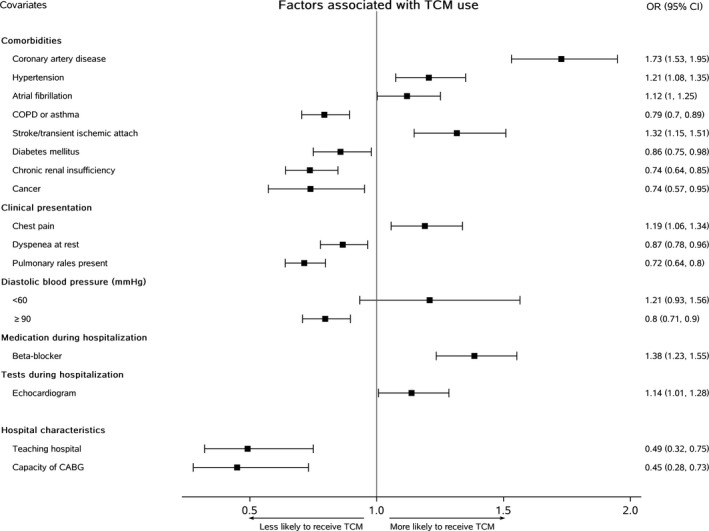
Patient‐ and hospital‐level characteristics associated with traditional Chinese medicine (TCM). CABG indicates coronary artery bypass grafting; COPD, chronic obstructive pulmonary disease; OR, odds ratio.

In hierarchical logistic regression models, some patient factors were strongly associated with TCM (Figure 4). Patients with coronary artery disease were more likely to receive TCM (OR, 1.73; 95% CI, 1.53–1.95), as were those with hypertension (OR, 1.21; 95% CI, 1.08–1.35) or stroke (OR, 1.32; 95% CI, 1.15–1.51). However, patients with chronic obstructive pulmonary disease (OR, 0.79; 95% CI, 0.70–0.89), diabetes mellitus (OR, 0.86; 95% CI, 0.75–0.98), chronic kidney disease (OR, 0.74; 95% CI, 0.64–0.85), and cancer (OR, 0.74; 95% CI, 0.57–0.95) were less likely to receive TCM. Patients presenting with chest pain were more likely to receive TCM (OR, 1.19; 95% CI, 1.06–1.34), whereas patients presenting with dyspnea at rest (OR, 0.87; 95% CI, 0.78–0.96) or pulmonary rales (OR, 0.72; 95% CI, 0.64–0.80) were less likely to receive TCM. Patients who received β blockers during hospitalization were more likely to receive TCM (OR, 1.38; 95% CI, 1.23–1.55); other evidenced‐based therapies were not significantly associated with TCM use.

We observed substantial variation in the use of TCM across hospitals (median OR, 3.29; 95% CI, 2.82–3.76), suggesting that the average odds of a patient receiving TCM at one random hospital versus another varied 3‐fold after adjusting for patient and treatment characteristics. Approximately 32% of the variance in use of TCM among patients was explained by the nested effect (or clustering) of patients among hospitals (intraclass correlation, 0.32). Patients treated in teaching hospitals (OR, 0.49; 95% CI, 0.32–0.75) or hospitals with capability of coronary artery bypass grafting (OR, 0.45; 95% CI, 0.28–0.73) were less likely to receive TCM.

### Association of TCM With Inpatient Outcomes

In fully adjusted hierarchical models, there was no association with TCM use and inpatient bleeding or with combination of mortality or treatment withdrawal (Table 4). We observed a significant increase in the risk for bleeding (OR, 1.39; 95% CI, 1.03–1.88) and death (OR, 1.36; 95% CI, 1.04–1.79) during hospitalization with Salvia miltiorrhiza (Table S2, Figure S2). The risk of bleeding was significantly higher in patients who received at least 2 days of Salvia miltiorrhiza therapy (Table S3). The risk of death was significantly higher in patients receiving Salvia miltiorrhiza for up to 9 days; however, mortality was not significantly higher in patients who received >9 days of Salvia miltiorrhiza therapy. In addition, the use of antiplatelets or anticoagulants with and without Salvia miltiorrhiza was not associated with greater in‐hospital bleeding or death (Table S4).

**Table 4:** Hospital Events Stratified by Use of TCM During Hospitalization

Variable	Overall		TCM		No TCM		*P* Value	Adjusted OR for any TCM	95% CI
	n	%	n	%	n	%			
All	9909	…	…	…	…	…	…	…	…
In‐hospital bleeding	255	2.6	167	2.3	88	3.5	0.0006	1.11	(0.86–1.45)
In‐hospital mortality or treatment withdrawal	342	3.4	242	3.3	100	4.0	0.0898	1.08	(0.84–1.39)

OR indicates odds ratio; TCM, traditional Chinese medicine.

## Discussion

In this nationally representative study of patients with HF admitted to Western medicine hospitals in China, three fourths of patients received TCM during HF hospitalization, and TCM was used in nearly all of the hospitals. One third of the agents used were prescribed for only 1 day during hospitalization, and only 12% of patients were prescribed TCM on discharge, in contrast with the longer durations of therapy studied in clinical trials. We did not find evidence that TCM was being used in lieu of evidence‐based therapies; instead, our findings suggest that TCM is used as a complementary therapy in the management of HF. Variation in use of TCM could be explained, in part, by patient‐ and hospital‐level factors, with ≈32% of the variation driven by hospital characteristics. Specifically, patients with statistically identical demographics and clinical findings on presentation and who received similar evidence‐based treatments for HF, on average, were ≈3 times more likely to receive TCM at some hospitals versus others. Finally, we observed a signal for increased bleeding and mortality with Salvia miltiorrhiza; although bleeding with this TCM has previously been described,^22^ the increase in mortality was an unexpected finding, warranting further investigation.

Our findings extend prior literature by demonstrating the frequent use of TCM among patients hospitalized with HF in a nationally representative and large sample. A previous national study observed TCM was used in the short‐term treatment of acute myocardial infarction in nearly all Western medicine hospitals throughout China, and most patients hospitalized with acute myocardial infarction received TCM.^2^ Our study demonstrates that TCM use was also prevalent in the clinical management of patients hospitalized with HF. Several reasons may account for its prevalence. First, treatment with TCM for HF is an ancient tradition in China that can be traced back to >1000 years. For many diseases, TCM combined with Western medicine is well accepted by physicians and patients.^23^ Second, TCM is a fully institutionalized part of health care in China.^24^ Specifically, the Chinese government encourages the use of TCM, as well as research about the efficacy and safety of TCM. Most of the TCMs, including both oral and intravenous types, are covered by the basic insurance provided by the government.^25^ To ensure the quality of TCM, the China Food and Drug Administration has strengthened the monitoring of manufacturing and postmarket safety of TCM in recent years. As of 2016, TCM was prescribed to >70 million outpatients and >2.5 million inpatients, accounting for 14% to 20% of healthcare visits in China.^26^ Third, some studies show that TCM may improve cardiac function and symptoms of HF. A systematic review^8^ of 9 randomized controlled trials assessing TCMs containing Salvia miltiorrhiza/danshen, Panax notoginseng, and Gingko, the most commonly used TCMs in this study, revealed evidence to support improvement in ventricular remodeling and function. Still, more studies designed and powered to find differences in patient outcomes, including data on long‐term outcomes, are needed.

Given the increasing number of publications showing therapeutic effects of TCM for HF, we were interested in assessing whether TCM was prescribed according to the duration tested in the most recent clinical trials, which might indicate that prescribing physicians are early adopters of emerging evidence for TCM. However, we found that TCM was used for much shorter durations than tested in prior trials. Specifically, in the most recent well‐conducted randomized clinical trials (Table S5) assessing the effect of TCM for HF, a consistent duration of at least 4 weeks was tested. Yet, in our sample of contemporary HF admissions, the median duration of TCM treatment was only 6 days, despite the median length of stay being 9 days. In addition, among patients who received TCM, only two thirds were prescribed them for >1 day. These findings suggest that the use of TCM may be driven more by cultural and clinical norms for treating symptoms and disease, than by the existing published data supporting their efficacy. Further investigations exploring the underlying reasons guiding the decision to use TCM in patients with HF, as well as their duration of use, are needed.

Patients with comorbid coronary artery disease and those who presented with chest pain were more likely to be prescribed TCM. Previous studies have demonstrated benefits of TCM for treating ischemic disease, including improved angiographic coronary blood flow and reflow,^27^ decreased stent restenosis, and less angina.^28^ Similarly, many studies showed a beneficial effect of TCM in the treatment of hypertension^29^, ^30^ and stroke,^31^, ^32^ which may explain the more frequent use of TCM among patients with comorbid hypertension and stroke.

We examined TCM use at the hospital level, the first such report, to our knowledge. Our findings revealed significant heterogeneity in the proportion of patients receiving TCM during hospitalization across hospitals. In some hospitals, none of the patients received TCM, whereas in others, all of the patients hospitalized with HF received TCM. Nonteaching hospitals were more likely to use TCM than teaching hospitals. Similarly, hospitals located in the central and western regions, which are generally poorer and tend to be less westernized, were more likely to use TCM.

Several limitations should be considered in the interpretation of these findings. First, as HF often occurs in the context of other medical illnesses, it is unknown whether the TCMs used in this study were intended to treat HF, versus other comorbidities, such as coronary artery disease, hypertension, or stroke. However, none of the Chinese guidelines for these conditions recommends TCM as a standard therapy. Second, our approach to examining factors associated with TCM was based on data from a retrospective review of medical records, which may not capture the full spectrum of patient, physician, or hospital characteristics that might influence use of TCM. Third, we are not able to evaluate the effects of other active TCM ingredients that are not the primary ingredient. In abstracting TCMs from the medical charts, we classified TCMs according to the primary active ingredient only; we did not document the name brand, which would be needed to identify other active ingredients. Although it is plausible that these nonprimary active ingredients may have had some effect, this classification is consistent with other studies of TCMs.^2^, ^3^ Finally, our work examining associations of TCM with in‐hospital outcomes is exploratory. Doses and formulations of TCM were not standardized, and we may not have had adequate power to exclude a clinically important association. In addition, we cannot confirm that in‐hospital bleeding occurred after TCM use for all patients.

In conclusion, TCM was widely used among patients admitted for HF in Western medicine hospitals throughout China. Although nearly all hospitals in our sample used TCM, we observed that some hospitals were >3 times as likely to use TCM for patients admitted with HF than other hospitals. Given the overall high use of TCM, along with the signal for potential harm with the most commonly used TCM, there is an urgent need for the study of the safety and efficacy of TCM, along with standardized approaches for its use in clinical practice.

## Author Contributions

Drs Krumholz and Li designed the study and take responsibility for all aspects of it. Drs Yu and Spatz wrote the first draft of the article, with further contributions from Drs Masoudi, Lu, Schulz, Krumholz, and Li. Q. Tan and Dr Liu performed statistical analysis. All authors approved the final version of the article. The corresponding author attests that all listed authors meet authorship criteria and that no others meeting the criteria have been omitted. The lead author affirms that the article is an honest, accurate, and transparent account of the study being reported; that no important aspects of the study have been omitted; and that any discrepancies from the study as originally planned (and, if relevant, registered) have been explained. Patient and Public Involvement: This study is a retrospective study based on abstraction of medical records. Patients were not involved in the recruitment or conduct of the study. The study findings will not be disseminated directly to patients, although the findings will inform quality improvement initiatives in hospitals after the dissemination of the study results. The study does not include patient advisors.

## Sources of Funding

This project was supported by the National Key Technology R&D Program (grants 2015BAI12B02, 2017YFC1310801, and 2017YFC1310803) from the Ministry of Science and Technology of China, the Chinese Academy of Medical Sciences Innovation Fund for Medical Sciences (grant CIFMS 2016‐I2M‐2‐004), and the 111 Project from the Ministry of Education of China (grant B16005). The funder of the study, the Chinese government, had no role in the study design, data collection, data analysis, data interpretation, or writing of the report. The corresponding author had full access to all data in the study and had final responsibility for the decision to submit for publication.

## Disclosures

Dr Spatz works under contract with the Centers for Medicare and Medicaid Services to develop and maintain performance measures. Harlan Krumholz was a recipient of a research grant, through Yale, from Medtronic and the U.S. Food and Drug Administration to develop methods for post‐market surveillance of medical devices; was a recipient of a research grant with Medtronic and Johnson & Johnson, through Yale, to develop methods of clinical trial data sharing; was a recipient of a research agreement, through Yale, from the Shenzhen Center for Health Information for work to advance intelligent disease prevention and health promotion; collaborates with the National Center for Cardiovascular Diseases in Beijing; received payment from the Arnold & Porter Law Firm for work related to the Sanofi clopidogrel litigation and from the Ben C. Martin Law Firm for work related to the Cook IVC filter litigation; chairs a Cardiac Scientific Advisory Board for UnitedHealth; is a participant/participant representative of the IBM Watson Health Life Sciences Board; is a member of the Advisory Board for Element Science, the Advisory Board for Facebook, and the Physician Advisory Board for Aetna; and is the founder of Hugohealth, a personal health information platform. Dr Masoudi has a contract with the American College of Cardiology for his role as Chief Science Officer of the National Cardiovascular Data Registry. The remaining authors have no disclosures to report. Dr Schulz is a consultant for Hugo Health.

## Supporting information


**Table S1.** Traditional Chinese Medicines used in the management of heart failure and their potential cardiac effects.
**Table S2.** Independent Association of type of TCM with in‐patient outcomes.
**Table S3.** In‐hospital outcomes stratified by duration of Salvia miltiorrhiza therapy.
**Table S4.** In‐hospital outcomes stratified by in‐hospital use of antiplatelet or anticoagulant agents, and Salvia miltiorrhiza.
**Table S5.** Characteristics of the randomized controlled trials of TCM for heart failure.
**Figure S1.** Use of TCM on day n of admission.
**Figure S2.** Association of TCM type with in‐patient bleeding or combination of death and treatment withdrawal.
