# Supplementary material for: Traditional Chinese Medicine Use in the Treatment of Acute Heart Failure in Western Medicine Hospitals in China: Analysis From the China PEACE Retrospective Heart Failure Study
Source: J Am Heart Assoc. 2019 Jul 31;8(15):e012776. doi: 10.1161/JAHA.119.012776 (PMC6761625; doi:10.1161/JAHA.119.012776)
Supplement: Supplementary file 1 — Table S1. Traditional Chinese Medicines used in the management of heart failure and their potential cardiac effects. Table S2. Independent Association of type of TCM with in‐patient outcomes. Table S3. In‐hospital outcomes stratified by duration of Salvia miltiorrhiza therapy. Table S4. In‐hospital outcomes stratified by in‐hospital use of antiplatelet or anticoagulant agents, and Salvia miltiorrhiza. Table S5. Characteristics of the randomized controlled trials of TCM for heart failure. Figure S1. Use of TCM on day n of admission. Figure S2. Association of TCM type with in‐patient bleeding or combination of death and treatment withdrawal. [file JAH3-8-e012776-s001.pdf]

Supplemental Material

Table S1. Traditional Chinese Medicines used in the management of heart failure and their potential cardiac effects.

Herbal Medicine	Related Formulations	Cardiac Effects	
		Suggested Benefit	Potential Harm
Salvia miltiorrhiza; ginseng; ginseng rubra[1-3]	Danshen dripping pills; Danshen polyphenolate injection, etc	Cardiac cytoprotective; anti-oxidation; anti-inflammation; antithrombotic; vasorelaxation; may improve angina symptoms	May cause bleeding problems with warfarin, aspirin or other antiplatelet drugs; potentiates digoxin activity
Panax notoginseng[4-8]	Xueshuantong injection, Sanqi Tongshu Capsule, etc	May improve heart function; possible small reduction in high blood pressure; treatment of angina and coronary artery disease; a calcium ion channel antagonist in vascular tissue; dilation of coronary arteries	High blood pressure with overuse; platelet inhibitor; may cause bleeding problems with warfarin, aspirin or other antiplatelet drugs
Radiax astragali[9-14]	Qiliqiangxin capsule, Qishenyiqi Dripping Pills, etc	Heart function improvement and suppression of left ventricular reverse remodeling; anti-inflammation; anti-oxidation	Not found
Gingko[15-18]	Ginkgo leaf capsule; Ginkgo leaf extract and dipyridamole injection, etc	May improve circulatory flow without appreciatively affecting blood pressure; platelet inhibitor	Increased bleeding tendency; hemorrhagic stroke; may cause bleeding problems with warfarin, aspirin or other antiplatelet drugs
Safflower[19-23]	Safflower yellow injection; Safflower extraction, etc	Anti-atherogenesis; anti-inflammation; may attenuate myocardial ischemia and reverse vascular remodeling	Large amounts of safflower might slow blood clotting. May cause bleeding problems with warfarin, aspirin or other antiplatelet drugs

Table S2. Independent Association of type of TCM with in-patient outcomes.

Description	Salvia miltiorrhiza/ ginseng/ ginseng rubra				Panax notoginseng				Gingko				Radiax astragali			
	OR	LOR	UOR	P	OR	LOR	UOR	P	OR	LOR	UOR	P	OR	LOR	UOR	P
**Bleeding**																
Unadjusted	1.12	0.85	1.48	0.4297	0.79	0.47	1.32	0.361	0.47	0.22	1.02	0.0547	0.27	0.05	1.43	0.1244
Adjusted; #1	1.14	0.86	1.51	0.3749	0.80	0.47	1.35	0.3943	0.48	0.22	1.03	0.0589	0.27	0.05	1.46	0.1292
Adjusted; #2	1.18	0.88	1.59	0.2613	0.86	0.50	1.47	0.5723	0.50	0.23	1.10	0.085	0.30	0.05	1.66	0.1673
Adjusted; #3	1.28	0.96	1.72	0.0946	1.04	0.61	1.77	0.8874	0.53	0.24	1.14	0.1029	0.33	0.06	1.74	0.1911
Adjusted; #4	1.29	0.96	1.73	0.0885	1.06	0.62	1.79	0.8419	0.57	0.26	1.23	0.1523	0.32	0.06	1.67	0.175
Adjusted; #5	1.32	0.99	1.77	0.061	1.07	0.63	1.81	0.8112	0.62	0.29	1.33	0.2158	0.34	0.07	1.74	0.1944
Adjusted; #6	1.39	1.03	1.88	0.0301	1.15	0.67	1.97	0.6118	0.64	0.30	1.40	0.2637	0.37	0.07	1.93	0.2379
**Death or Withdrawal of Treatment**																
Unadjusted	1.11	0.85	1.44	0.4402	0.66	0.40	1.09	0.1026	0.51	0.26	1.02	0.0551	1.08	0.49	2.41	0.8464
Adjusted; #1	1.11	0.86	1.44	0.4263	0.66	0.40	1.09	0.103	0.51	0.26	1.00	0.0508	1.11	0.50	2.49	0.7944
Adjusted; #2	1.20	0.92	1.56	0.1718	0.77	0.47	1.26	0.2958	0.57	0.29	1.11	0.0989	1.03	0.46	2.34	0.9352
Adjusted; #3	1.26	0.97	1.64	0.0861	0.86	0.53	1.39	0.5301	0.58	0.30	1.12	0.1038	1.21	0.54	2.70	0.6472
Adjusted; #4	1.25	0.96	1.62	0.1007	0.86	0.53	1.39	0.5272	0.57	0.29	1.09	0.0909	1.25	0.56	2.81	0.5817
Adjusted; #5	1.31	1.00	1.71	0.0482	0.88	0.54	1.44	0.6145	0.57	0.30	1.12	0.1015	1.29	0.56	2.99	0.5455
Adjusted; #6	1.36	1.04	1.79	0.0257	0.92	0.56	1.51	0.7355	0.59	0.30	1.14	0.1154	1.30	0.56	3.03	0.547

Description	Safflower				Other TCM				≥2 TCM			
	OR	LOR	UOR	P	OR	LOR	UOR	P	OR	LOR	UOR	P

**Bleeding**												
Unadjusted	0.43	0.20	0.96	0.0383	0.53	0.26	1.08	0.0801	0.82	0.59	1.14	0.2325
Adjusted; #1	0.45	0.20	1.01	0.0522	0.52	0.26	1.07	0.0755	0.83	0.59	1.16	0.273
Adjusted; #2	0.51	0.23	1.16	0.1075	0.57	0.27	1.18	0.1264	0.92	0.65	1.30	0.642
Adjusted; #3	0.50	0.22	1.11	0.0895	0.68	0.33	1.39	0.2875	1.02	0.73	1.44	0.9017
Adjusted; #4	0.49	0.22	1.09	0.0784	0.67	0.33	1.36	0.2634	1.04	0.74	1.47	0.8192
Adjusted; #5	0.49	0.22	1.11	0.0859	0.66	0.32	1.35	0.2523	1.05	0.74	1.48	0.7877
Adjusted; #6	0.51	0.23	1.16	0.1064	0.72	0.35	1.48	0.3687	1.10	0.78	1.57	0.5799
**Death or Withdrawal of Treatment**												
Unadjusted	0.39	0.17	0.91	0.0303	0.36	0.17	0.79	0.0102	0.73	0.54	1.00	0.048
Adjusted; #1	0.41	0.17	0.95	0.0383	0.35	0.16	0.77	0.0085	0.72	0.53	0.98	0.0362
Adjusted; #2	0.47	0.21	1.08	0.076	0.39	0.18	0.81	0.0122	0.88	0.65	1.20	0.4254
Adjusted; #3	0.50	0.22	1.13	0.0961	0.43	0.21	0.89	0.022	0.99	0.73	1.34	0.9409
Adjusted; #4	0.50	0.22	1.12	0.0922	0.43	0.21	0.89	0.0231	0.99	0.72	1.34	0.9231
Adjusted; #5	0.49	0.21	1.12	0.0904	0.45	0.21	0.94	0.0326	1.05	0.77	1.43	0.777
Adjusted; #6	0.50	0.22	1.15	0.1041	0.46	0.22	0.97	0.0423	1.07	0.78	1.47	0.6938

#1 -- Demographics (sex, age, insurance)

#2 -- #1 and admission presentation (symptoms, signs, ejection fraction, Admission to ICU/CCU, NYHA functional class)

#3 -- #2 and comorbidities (coronary heart disease, hypertension, diabetes mellitus, Dyslipidemia, etc).

#4 -- #3 and diagnosis tests (echocardiography, imaging, BNP/NT-ProBNP)

#5 -- #4 and Medications used during hospitalization (ACEI/ARB, beta blocker, aldosterone antagonist, anticoagulant, antiplatelet).

#6 -- #5 and hospital characteristics (level, region, CABG capability, etc)

Table S3. In-hospital outcomes stratified by duration of Salvia miltiorrhiza therapy.

**Duration of Salvia miltiorrhiza therapy (days)**	**Overall**	**In-patient bleeding**		**p**	**Adjusted OR**		**p**
		n	%		OR	95% CI	
0	2514	88	3.5	0.036	reference		0.001
1	928	16	1.7		0.84	0.52-1.35	
2-9	1138	34	3.0		1.46	1.01-2.12	
>9	843	32	3.8		1.99	1.36-2.92	

**Duration of Salvia miltiorrhiza therapy (days)**	**Overall**	**In-patient mortality**		**p**	**Adjusted OR**		**p**
		n	%		OR	95% CI	
0	2514	77	3.1	0.189	reference		<.001
1	928	36	3.9		2.33	1.54-3.54	
2-9	1138	42	3.7		1.64	1.13-2.37	
>9	843	19	2.3		1.04	0.65-1.67	

Table S4. In-hospital outcomes stratified by in-hospital use of antiplatelet or anticoagulant agents, and Salvia miltiorrhiza.

**Antiplatelet or anticoagulant agents**	**Salvia miltiorrhiza**	**Overall**	**In-patient bleeding**		**p**	**In-patient mortality**		**p**
			n	%		n	%	
Yes	Yes	2166	67	3.1	0.085	68	3.1	0.961
	No	1671	69	4.1		52	3.1	
No	Yes	913	21	2.3	0.948	35	3.8	0.317
	No	843	19	2.2		25	3.0	

Table S5 Characteristics of the randomized controlled trials of TCM for heart failure.

Studies included	Clinical diagnosis	Patients (n)	Treatment comparison	Follow-up (weeks)	Endpoints	Efficacy	Safety (adverse events)	Jadad score
Zou X, et al. 2006[24]	CHF with NYHA class of II to III	100	*Nuanxin* capsule versus placebo	52	Effective response; NYHA functional classification; drug-related side effects	Positive	Yes (0/50 vs. 1/50)	3
Zou X, et al. 2011[25]	CHF with NYHA class of II to III	150	*Nuanxin* capsule versus placebo for 24w	24	Effective response; NYHA functional classification; rehospitalization; acute heart failure; death; drug-related side effects	Positive	Yes (1/71 vs. 0/73)	5
Miao JH, et al. 2012[26]	CHF with NYHA class of II to III	200	*Yiqihuayu* capsule versus placebo for 8w	8	NYHA functional classification; the frequency of patients with LVEF>40%; drug-related side effects	Positive	Yes (0 vs. 0)	3
Wang C, et al. 2012[27]	CHF with NYHA class of II to III	280	*Shencaotongmai* granule versus placebo for 12w	12	Effective response; NYHA functional classification; LVEF; drug-related side effects	Positive	Yes (1/140 vs. 1/140)	5
Zhang Y, et al. 2012[28]	CHF with NYHA class of II to III	280	*Qiangxintongmai* granule versus placebo for 12w	12	6MWD	Positive	NA	4
Li XL, et al. 2013[29]	CHF with NYHA class of II to IV	512	*Qiliqiangxin* capsule versus placebo for 12w	12	NYHA functional classification; LVEF; 6MWD; reduction in NT-proBNP; composite cardiac events; drug-related adverse events	Positive	Yes (20/250 vs. 23/250)	5
Fu XX, et	CHF with	140	Hot compress	12	Effective response; NYHA functional	Positive	NA	4

al. 2014[30]	NYHA class of II to IV		with *Zhuangshenling* formula versus placebo for 12w		classification; 6MWD; BNP			
Su XH, et al. 2014[31]	CHF with NYHA class of II to III	100	*Yangxin* decoction versus placebo	4	Effective response; NYHA functional classification; physical examination	Positive	NA	3
Xian SX, et al. 2015[32]	CHF with NYHA class of II to III	228	*Yangxinkang* tablet versus placebo for 4w	4	Effective response; NYHA functional classification; physical examination; drug-related side effects	Positive	Yes (0 vs. 0)	5
Wang X, et al. 2017[33]	CHF with NYHA class of II to III	465	*Shensong Yangxin capsule* versus placebo for 12w	12	Change of number of VPCs; NYHA functional classification; LVEF; NT-proBNP; MLHFQ	Positive	Yes (5/232 vs. 8/233)	5

We included reports of clinical studies published form January 1st, 2006 to September 4th, 2018 with the following criteria: 1) study patients with a definite diagnosis of heart failure who were randomized to receive TCM, contemporary medication, or placebo; 2) sample size in each study group ≥50 cases; 3) follow-up in each study group ≥4 weeks; and 4) quantitative measurements of surrogate endpoints and/or adverse cardiovascular events and/or adverse drug effects available to facilitate outcome analysis. We excluded reports of studies with the following features: 1) studies were nonrandomized and/or non–double-blinded; 2) patients enrolled had no definite diagnosis; 3) studies compared different TCM medications; and 4) studies reported only symptomatic changes of patients, without objective laboratory measurements or physical examination. Methodological quality was evaluated for each study with a Jadad score between 0 (weakest) to 5 (strongest), as described previously (6); any study with a Jadad score <3 was considered to be of poor quality and was excluded. In addition, when 2 papers reported the results of the same study, the paper with less data was excluded.

ACEI, angiotensin converting enzyme inhibitors; ARB, angiotensin-receptor blockers; BNP, brain natriuretic peptide; CAD, coronary artery disease; CCB, calcium channel blockers; CHF, chronic heart failure; LVEF, left ventricular ejection fraction; 6MWD, 6-min walking distance; NA, not available; NT-proBNP, N-terminal prohormone of brain natriuretic peptide; NYHA, New York Heart Association; VPC, ventricular premature complexes; MLHFQ, Minnesota Living with Heart Failure Questionnaire.

Figure S1. Use of TCM on day n of admission.

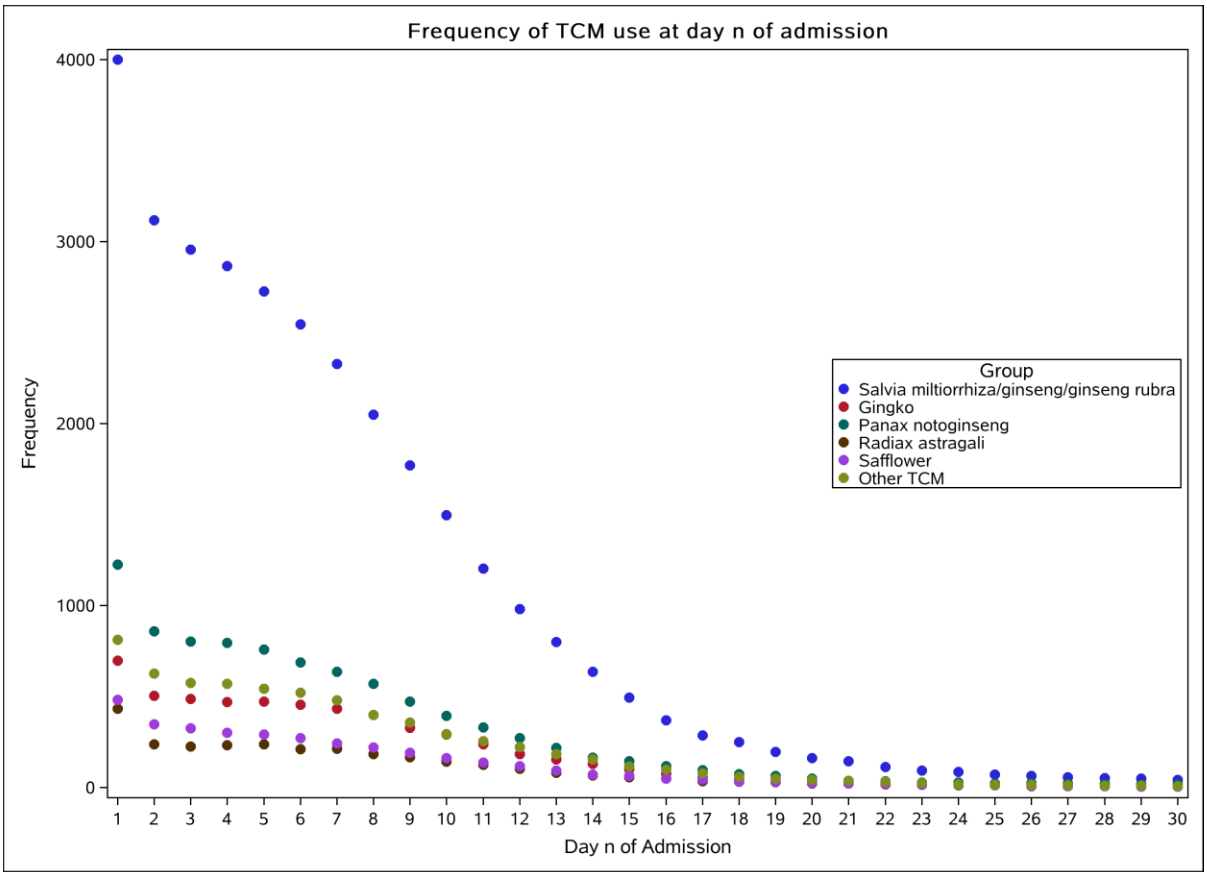

Figure S2. Association of TCM type with in-patient bleeding or combination of death and treatment withdrawal.

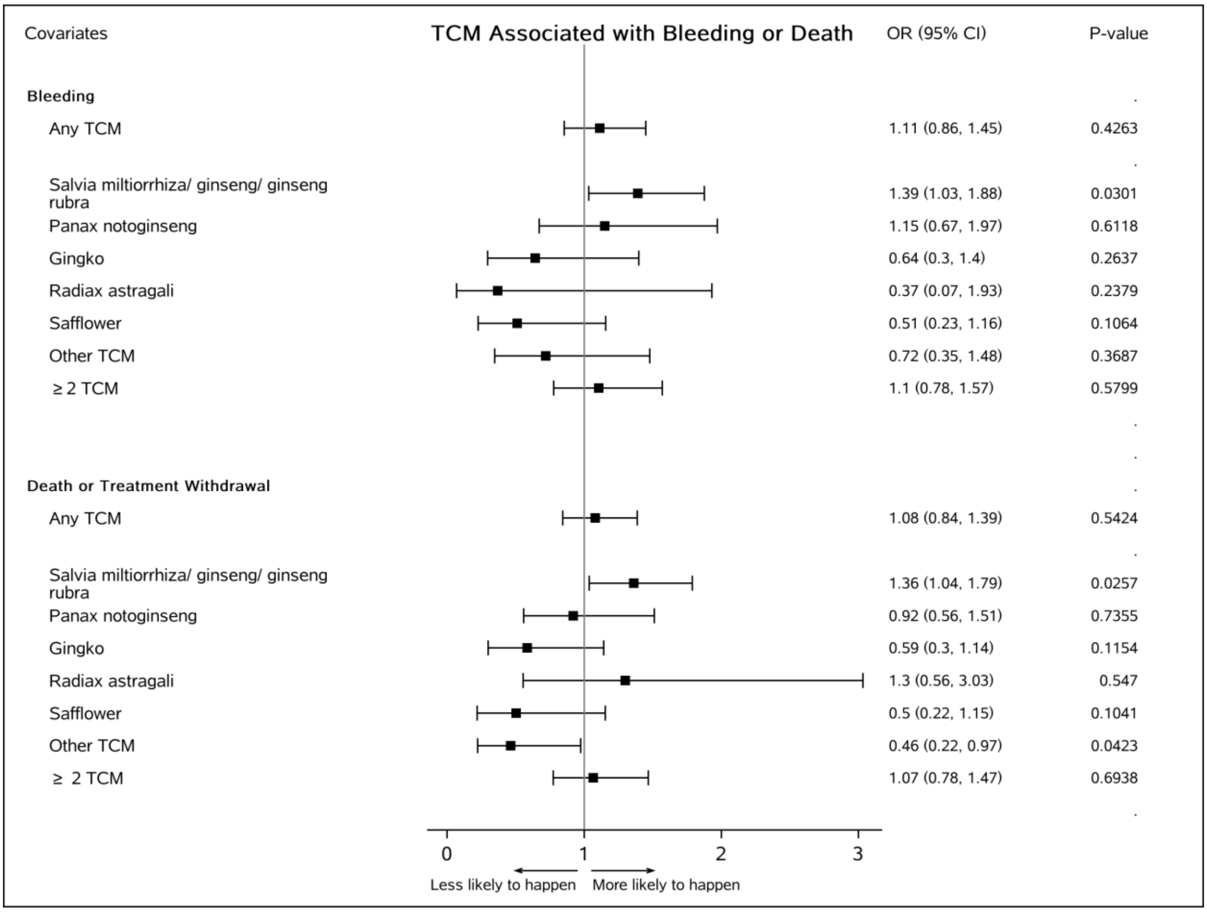

* Individual type of TCM represent patients use and only use this type of TCM.

Supplemental References:

1. Jian-Ming L, Qizhi Y and Changyi C. Ginseng Compounds: An Update on their Molecular Mechanisms and Medical Applications. *Current Vascular Pharmacology*. 2009;7:293-302.
2. Gao S, Liu Z, Li H, Little PJ, Liu P and Xu S. Cardiovascular actions and therapeutic potential of tanshinone IIA. *Atherosclerosis*. 2012;220:3-10.
3. Cheng TO. Cardiovascular effects of Danshen. *International Journal of Cardiology*. 2007;121:9-22.
4. Zhang YG, Zhang HG, Zhang GY, Fan JS, Li XH, Liu YH, Li SH, Lian XM and Tang Z. Panax notoginseng saponins attenuate atherosclerosis in rats by regulating the blood lipid profile and an anti-inflammatory action. *Clinical and Experimental Pharmacology & Physiology*. 2008;35:1238-44.
5. Chen S, Liu J, Liu X, Fu Y, Zhang M, Lin Q, Zhu J, Mai L, Shan Z, Yu X, Yang M and Lin S. Panax notoginseng saponins inhibit ischemia-induced apoptosis by activating PI3K/Akt pathway in cardiomyocytes. *Journal of Ethnopharmacology*. 2011;137:263-70.
6. Fan JS, Liu DN, Huang G, Xu ZZ, Jia Y, Zhang HG, Li XH and He FT. Panax notoginseng saponins attenuate atherosclerosis via reciprocal regulation of lipid metabolism and inflammation by inducing liver X receptor alpha expression. *Journal of Ethnopharmacology*. 2012;142:732-8.
7. Pan C, Huo Y, An X, Singh G, Chen M, Yang Z, Pu J and Li J. Panax notoginseng and its components decreased hypertension via stimulation of endothelial-dependent vessel dilatation. *Vascular Pharmacology*. 2012;56:150-8.

8. Xu D, Huang P, Yu Z, Xing DH, Ouyang S and Xing G. Efficacy and Safety of Panax notoginseng Saponin Therapy for Acute Intracerebral Hemorrhage, Meta-Analysis, and Mini Review of Potential Mechanisms of Action. *Frontiers in Neurology*. 2014;5:274.

9. Lu S, Chen KJ, Yang QY and Sun HR. Progress in the research of Radix Astragali in treating chronic heart failure: Effective ingredients, dose-effect relationship and adverse reaction. *Chinese Journal of Integrative Medicine*. 2011;17:473-7.

10. Jia Y, Zuo D, Li Z, Liu H, Dai Z, Cai J, Pang L and Wu Y. Astragaloside IV Inhibits Doxorubicin-Induced Cardiomyocyte Apoptosis Mediated by Mitochondrial Apoptotic Pathway <i>via</i> Activating the PI3K/Akt Pathway. *Chemical and Pharmaceutical Bulletin*. 2014;62:45-53.

11. Lu J, Zou D and Zhang J. Preventive effect of radix Astragali on insulin resistance caused by tumor necrosis factor-alpha. *Chinese Journal of Integrated Traditional and Western Medicine*. 1999;19:420-2.

12. Li L, Hou X, Xu R, Liu C and Tu M. Research review on the pharmacological effects of astragaloside IV. *Fundamental & Clinical Pharmacology*. 2017;31:17-36.

13. Chen W, Zhang YY, Wang Z, Luo XH, Sun WC and Wang HB. Phenolic derivatives from Radix Astragali and their anti-inflammatory activities. *Natural Product Communications*. 2014;9:1577-80.

14. Shahzad M, Shabbir A, Wojcikowski K, Wohlmuth H and Gobe GC. The Antioxidant Effects of Radix Astragali (Astragalus membranaceus and Related Species) in Protecting Tissues from Injury and Disease. *Current Drug Targets*. 2016;17:1331-40.

15. Dutta-Roy AK, Gordon MJ, Kelly C, Hunter K, Crosbie L, Knight-Carpentar T and

Williams BC. Inhibitory effect of Ginkgo biloba extract on human platelet aggregation. *Platelets*. 1999;10:298-305.

16. Heck AM, DeWitt BA and Lukes AL. Potential interactions between alternative therapies and warfarin. *American journal of health-system pharmacy : AJHP : official journal of the American Society of Health-System Pharmacists*. 2000;57:1221-7; quiz 1228-30.

17. Bent S, Goldberg H, Padula A and Avins AL. Spontaneous bleeding associated with ginkgo biloba: a case report and systematic review of the literature: a case report and systematic review of the literature. *Journal of General Internal Medicine*. 2005;20:657-61.

18. Tian J, Liu Y and Chen K. Ginkgo biloba Extract in Vascular Protection: Molecular Mechanisms and Clinical Applications. *Curr Vasc Pharmacol*. 2017;15:532-548.

19. Bao LD, Wang Y, Ren XH, Ma RL, Lv HJ and Agula B. Hypolipidemic effect of safflower yellow and primary mechanism analysis. *Genetics and Molecular Research : GMR*. 2015;14:6270-8.

20. Liu Y, Tian X, Cui M and Zhao S. Safflower yellow inhibits angiotensin II-induced adventitial fibroblast proliferation and migration. *Journal of Pharmacological Sciences*. 2014;126:107-14.

21. Takimoto T, Suzuki K, Arisaka H, Murata T, Ozaki H and Koyama N. Effect of N-(p-coumaroyl)serotonin and N-feruloylserotonin, major anti-atherogenic polyphenols in safflower seed, on vasodilation, proliferation and migration of vascular smooth muscle cells. *Molecular Nutrition & Food Research*. 2011;55:1561-71.

22. Wang CC, Choy CS, Liu YH, Cheah KP, Li JS, Wang JT, Yu WY, Lin CW, Cheng HW and Hu CM. Protective effect of dried safflower petal aqueous extract and its main constituent,

carthamus yellow, against lipopolysaccharide-induced inflammation in RAW264.7 macrophages. *Journal of the science of food and agriculture*. 2011;91:218-25.

23. Zhou MX, Fu JH, Zhang Q and Wang JQ. Effect of hydroxy safflower yellow A on myocardial apoptosis after acute myocardial infarction in rats. *Genetics and Molecular research : GMR*. 2015;14:3133-41.

24. Zou X, Pan G, Liu Z, Lin X and Mai S. Clinical Observation of Nuanxin Capsule on 50 Patients with Chronic Heart Failure of Blood Stasis Due to Deficiency of Qi Syndrome. *Liaoning Journal of Traditional Chinese Medicine*. 2006.

25. Zou X, Pan GM and Sheng XG. Double blinded randomized and controlled study on treatment of chronic heart failure by nuanxin capsule. *Chinese Journal of Integrated Traditional and Western Medicine*. 2011;31:19-22.

26. Miao J and Li Y. Clinical observation of Yiqihuayu capsule in the treatment of 100 patients with Qi deficiency and blood stasis due to chronic systolic heart failure. *J Shandong Univ Trad Chin Med*. 2012:205-207.

27. Wang C, Zhang Y and Gong LH. Treatment of chronic heart failure by shencao tongmai granule: a multi-centered, double-blinded, randomized, parallel controlled trial. *Chinese Journal of Integrated Traditional and Western Medicine*. 2012;32:612-5.

28. Zhang Y, Gong LH, Fan L, Wang C and Liao JD. A randomized clinical trial: effect of Qi-reinforcing-blood-activating drugs on life quality in patients with chronic heart failure. *Chinese Archives of Traditional Chinese Medicine*. 2012;30:1193-1195.

29. Li X, Zhang J, Huang J, Ma A, Yang J, Li W, Wu Z, Yao C, Zhang Y, Yao W, Zhang B and Gao R. A multicenter, randomized, double-blind, parallel-group, placebo-controlled study of

the effects of qili qiangxin capsules in patients with chronic heart failure. *Journal of the American College of Cardiology*. 2013;62:1065-72.

30. Fu XX, Lu J, Yang F and Xiao WJ. Treatment of chronic heart failure of Xin-Shen yang deficiency, interior retention of water-fluid syndrome by external application of Zhuangshenling recipe combined with western medicine: a clinical study. *Chinese Journal of Integrated Traditional and Western Medicine*. 2014;34:808-11.

31. Su X, Wang S and Mi D. To observe the effect of Yangxin Decoction in the treatment of chronic congestive heart failure. *Chinese Medicine Modern Distance Education of China*. 2014.

32. Xian SX, Yang ZQ, Ren PH, Ye XH, Ye SL, Wang QH, Wang ZH, Shen SJ and Huang XW. Effect of yangxinkang tablets on chronic heart failure: A multi-center randomized double-blind placebo-controlled trial. *Chinese Journal of Integrative Medicine*. 2015;21:733-42.

33. Wang X, Hu D, Dang S, Huang H, Huang CX, Yuan MJ, Tang YH, Zheng QS, Yin F, Zhang S, Zhang BL and Gao RL. Effects of Traditional Chinese Medicine Shensong Yangxin Capsules on Heart Rhythm and Function in Congestive Heart Failure Patients with Frequent Ventricular Premature Complexes: A Randomized, Double-blind, Multicenter Clinical Trial. *Chinese Medical Journal*. 2017;130:1639-1647.
